# Supplementary material for: Transient non-Hermitian skin effect
Source: Nat Commun. 2022 Dec 12;13:7668. doi: 10.1038/s41467-022-35448-2 (PMC9744917; doi:10.1038/s41467-022-35448-2)
Supplement: Supplementary file 1 — Supplementary information [file 41467_2022_35448_MOESM1_ESM.pdf]

Supplementary Information for "Transient non-Hermitian skin effect"

Zhongming Gu[1], He Gao[2⋆], Haoran Xue[3⋆], Jensen Li[4], Zhongqing Su[2] and Jie Zhu[1⋆]

[1]Institute of Acoustics, School of Physics Science and Engineering, Tongji University, Shanghai 200092, China.
[2]Department of Mechanical Engineering, The Hong Kong Polytechnic University, Hung Hom, Kowloon, Hong Kong SAR, China.
[3]Division of Physics and Applied Physics, School of Physical and Mathematical Sciences, Nanyang Technological University, Singapore 637371, Singapore.
[4]Department of Physics, The Hong Kong University of Science and Technology, Kowloon, Hong Kong, China.
⋆e-mail: h.e.gao@connect.polyu.hk; haoran001@e.ntu.edu.sg; jiezhu@tongji.edu.cn.

Note 1. Ring resonator lattice model.

In this section, we present detailed derivations for the ring resonator model using the transfer matrix approach. Figure S1 illustrates one unit cell with periodic boundary condition, in which (a_1, a_2, b_1, b_2) denotes the complex amplitudes at the according parts. The corresponding scattering equations can be expressed as:

$$\begin{bmatrix} b_1 e^{-i\varphi_b/2-r_1} \\ a_1 e^{-i\varphi_a/2-r_0} \end{bmatrix} = S \begin{bmatrix} b_2 \\ a_2 \end{bmatrix}, \tag{S1}$$

$$\begin{bmatrix} b_2 e^{-i\varphi_b/2-r_2} \\ a_2 e^{-i\varphi_a/2-r_0+ik_x} \end{bmatrix} = S \begin{bmatrix} b_1 \\ a_1 e^{ik_x} \end{bmatrix}. \tag{S2}$$

S is the coupling matrix, which can be described as:

$$S = \begin{bmatrix} S_{11} & S_{12} \\ S_{21} & S_{22} \end{bmatrix} = \begin{bmatrix} \cos\theta & -i\sin\theta \\ -i\sin\theta & \cos\theta \end{bmatrix}. \tag{S3}$$

From equation (S1), we obtain

$$\begin{bmatrix} b_1 \\ b_2 \end{bmatrix} = \begin{bmatrix} e^{i\varphi_b/2-i\varphi_a/2-r_0+r_1} S_{11}/S_{21} & e^{(i\varphi_b/2+r_1)}(S_{12}-S_{11}S_{22}/S_{21}) \\ e^{-(i\varphi_a/2+r_0)}/S_{21} & -S_{22}/S_{21} \end{bmatrix} \begin{bmatrix} a_1 \\ a_2 \end{bmatrix} = T_1 \begin{bmatrix} a_1 \\ a_2 \end{bmatrix}. \tag{S4}$$

From equation (S2), we can have:

$$e^{ik_x} \begin{bmatrix} a_1 \\ a_2 \end{bmatrix} = \begin{bmatrix} -S_{11}/S_{12} & e^{(-i\varphi_b-r_2)}/S_{12} \\ e^{(i\varphi_a/2+r_0)}(S_{21}-S_{11}S_{22}/S_{12}) & e^{(i\varphi_a/2-i\varphi_b/2+r_0-r_2)} S_{22}/S_{12} \end{bmatrix} \begin{bmatrix} b_1 \\ b_2 \end{bmatrix} = T_2 \begin{bmatrix} b_1 \\ b_2 \end{bmatrix}. \tag{S5}$$

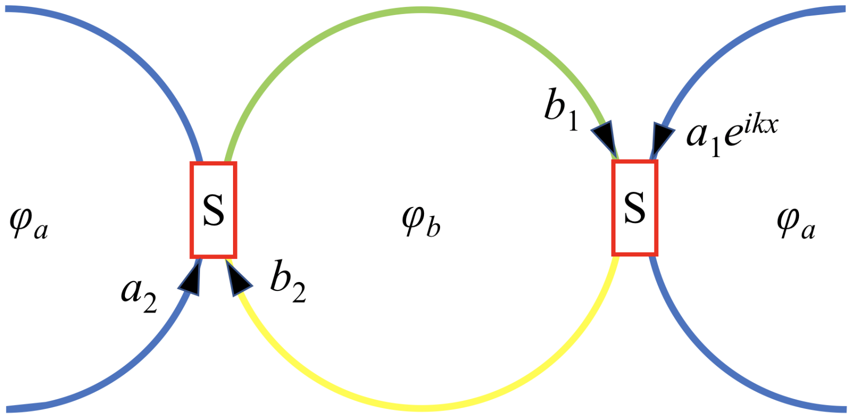

Fig. S1 Illustration of one unit cell with periodic boundary condition. The loss/gain parameters for the site ring (in blue), upper half part (in green) and lower half part (in yellow) of the link ring are $2r_0, r_1$ and r_2, respectively. φ_a and φ_b are the round trip phases in the site and link rings.

By combining equation (S4) and equation (S5), we have:

$$e^{ik_x}\begin{bmatrix}a_1\\a_2\end{bmatrix} = T_2T_1\begin{bmatrix}a_1\\a_2\end{bmatrix} = T\begin{bmatrix}a_1\\a_2\end{bmatrix}. \tag{S6}$$

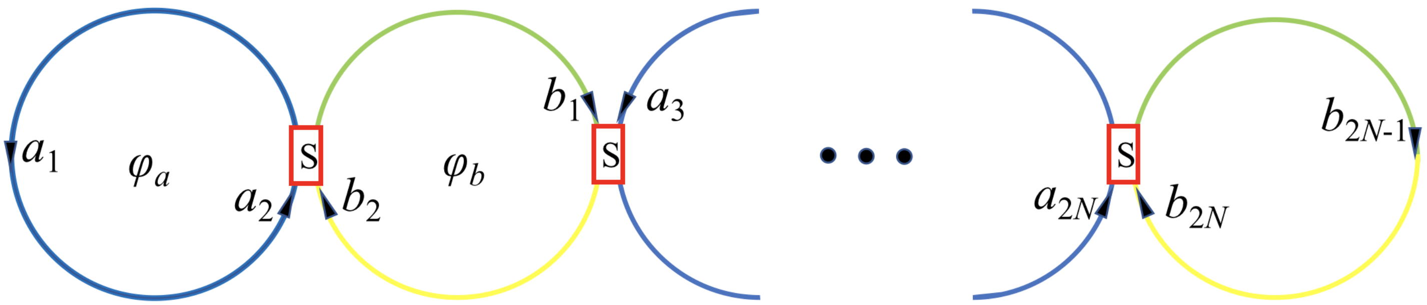

Fig. S2 Illustration of the coupled ring lattice composed of N unit cells.

For a finite chain system with N unit cells, as shown in Fig. S2. The according field amplitudes can be described by $(a_1, a_2, b_1, b_2, \cdots, a_{2N-1}, a_{2N}, b_{2N-1}, b_{2N})$. Based on equation (S6), we can obtain:

$$\begin{bmatrix}a_{2N-1}\\a_{2N}\end{bmatrix} = T\begin{bmatrix}a_{2N-3}\\a_{2N-2}\end{bmatrix} = TT\begin{bmatrix}a_{2N-5}\\a_{2N-4}\end{bmatrix} = T^{N-1}\begin{bmatrix}a_1\\a_2\end{bmatrix} = \begin{bmatrix}t_{11} & t_{12}\\t_{21} & t_{22}\end{bmatrix}\begin{bmatrix}a_1\\a_2\end{bmatrix}. \tag{S7}$$

In the first unit cell, we have:

$$a_2 = a_1 e^{(i\varphi_a/2+r_0)} = A_1 a_1. \tag{S8}$$

Besides, in the last unit cell, we have:

$$\begin{gathered}\begin{bmatrix}b_{2N-1}e^{-(i\varphi_b/2+r_1)}\\a_{2N-1}e^{-(i\varphi_a/2+r_0)}\end{bmatrix} = S\begin{bmatrix}b_{2N}\\a_{2N}\end{bmatrix},\\ b_{2N} = b_{2N-1}e^{(i\varphi_b/2+r_2)}.\end{gathered} \tag{S9}$$

From equation (S9), we obtain:

$$\begin{aligned}a_{2N} &= \frac{e^{-(i\varphi_a/2+r_0)}(e^{-(i\varphi_b+r_1+r_2)} - S_{11})}{S_{22}e^{-(i\varphi_b+r_1+r_2)} + S_{12}S_{21} - S_{11}S_{22}}a_{2N-1}\\ &= A_2 a_{2N-1}.\end{aligned} \tag{S10}$$

Then from equation (S7), (S8) and (S10), we can have:

$$A_2(t_{11} + A_1 t_{12}) = t_{21} + A_1 t_{22}. \tag{S11}$$

By solving equation (S6) and (S11), the eigen spectra under periodic boundary condition and open boundary condition can be obtained, respectively.

Note 2. Non-local response with the complex-frequency excitation.

It has been shown in active systems that the response of NHSE is stronger as the excitation is farther from the boundary. In this section, we show that this unique non-local response feature can be observed using the complex-frequency excitation in passive systems. To this end, we consider a finite-sized Hatano-Nelson chain with 31 sites, as illustrated in Fig. S3. Other lattice parameters are the same as the ones in the main text. We place the excitation at three different positions and the amplitude profiles at $t = 20$ are shown in Fig. S3. As can be seen, the non-local response is quite clearly present.

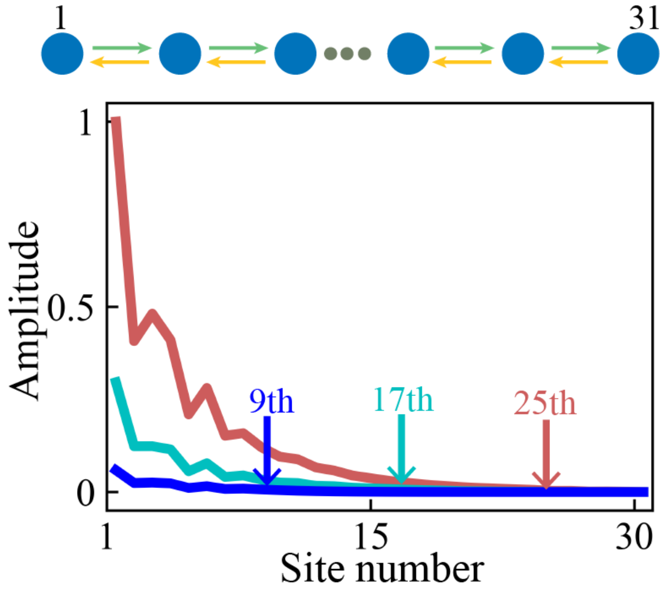

Fig. S3 The amplitude profile of the finite lattice at $t = 20$ under the excitation at different positions. The probed signals are normalized to the excitation signal. The finite-sized lattice is composed of 31 units. The complex-frequency excitation signal is respectively injected to the 25th, 17th and 9th sites, and the captured responses are denoted by the red, cyan and blue lines, respectively.

Note 3. Design procedure of the acoustic model.

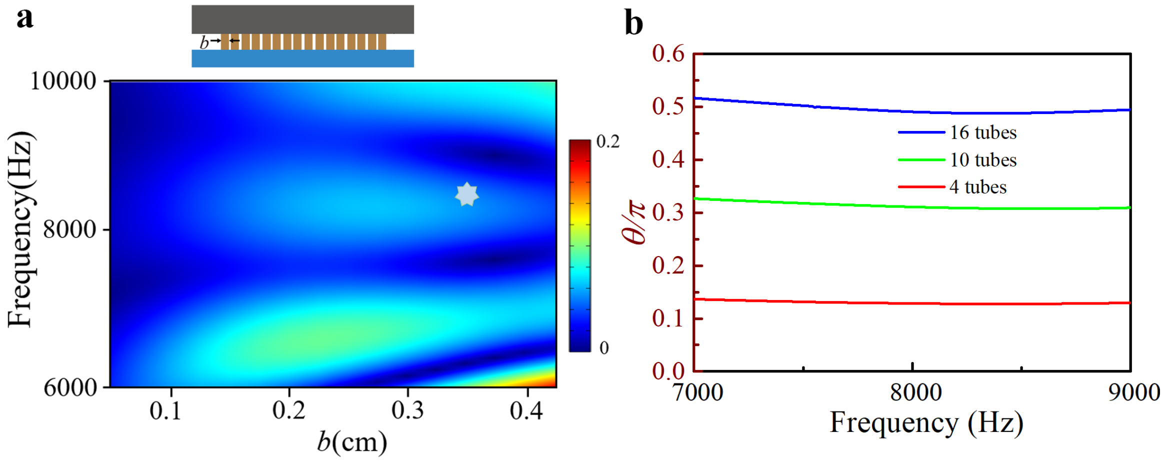

Fig. S4 The scattering properties of the coupling region. a The reflection property as a function of the structural parameter b and frequency. **b** The coupling angle with different numbers of tubes: 4 tubes, 10 tubes and 16 tubes.

There are two main considerations in designing the acoustic structure. The first one is that we want to achieve perfect coupling (i.e., full transmission) between two coupled ring resonators. This is for the purpose of clear visualization of the NHSE (the propagation path can be easily visualized in the perfect coupling case). The second consideration is the minimization of the reflection during the coupling process. This is crucial since our theory is based on the assumption that the clockwise and counterclockwise modes are decoupled.

To obtain optimal structural parameters that fulfill above requirements, we consider the structure shown in top panel in Fig. S4a, which consists of two acoustic waveguides coupled by small tubes with fixed spacings (0.47 cm) and widths (0.7 cm). First, we sweep the thickness of the tubes (denoted by b) and frequency for a structure with 16 tubes to determine the value of parameter b and working frequency range with near zero reflection. According to the numerical results shown in bottom panel in Fig. S4a, we adopt b=0.35 cm and working frequency around 8250 Hz (denoted by the grey star in Fig. S4a). Then, the coupling strength can be varied by changing the number of tubes in the coupling region. As shown in Fig. S4b, 16 tubes indeed achieve perfect coupling in the frequency range of our interest, compared to the cases with 4 tubes and 10 tubes.

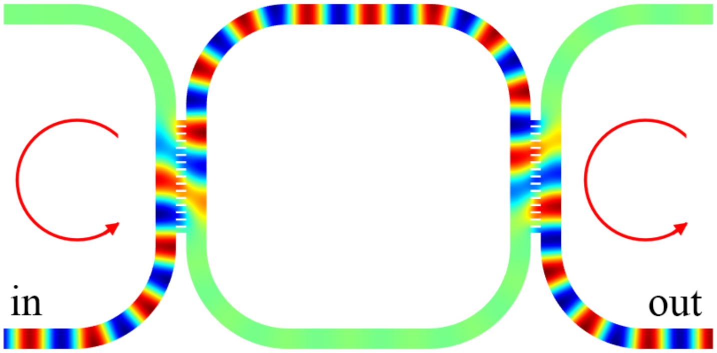

Fig. S5Acoustic wave propagation through one unit cell.

To check the performance of the design, we numerically calculated the pressure field at 8250 Hz of a basic unit cell to distinguish the modes with different circulations. As shown in Fig. S5, the mode injected from lower left side can be perfectly transmitted to the lower right side, without any noticeable reflection.

Note 4. Eigen spectra of the ring resonator lattice model.

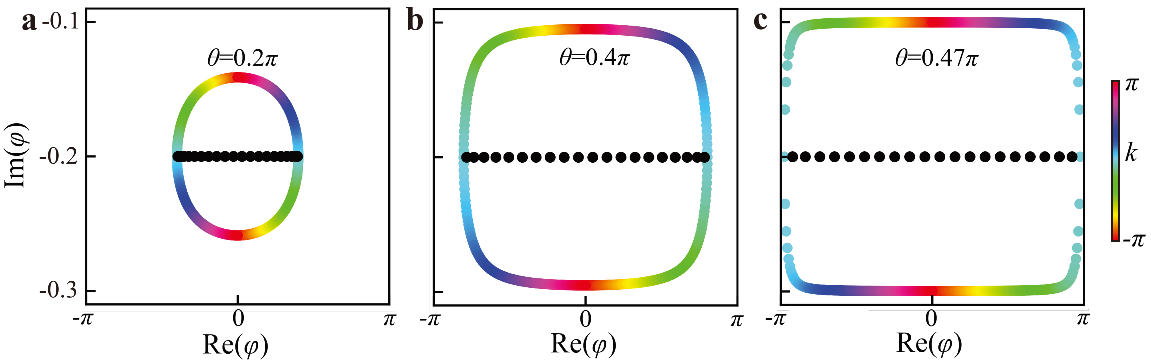

Fig. S6 The eigen spectra for the PBC and OBC lattices derived from the transfer matrix method with a θ=0.2π, b θ=0.4π and c θ=0.47π.

In this section, we show the PBC and OBC spectra of the ring resonator lattices for various values of the coupling strength θ. In the acoustic design, we optimize the structural parameters to obtain the perfect coupling (i.e., $\theta = 0.5\pi$) for a better visualization of NHSE, which also gives rise to the straight lineshape in the PBC spectrum that is distinct from the closed loop spectrum in conventional NHSE (for example, see Fig. 2b in the main text). As θ decreases, the two straight lines in the PBC spectrum approach each other and finally form a single closed loop, as cases given in Fig. S6a–c. During this process, the width of the OBC spectrum keeps decreasing, make it within the loop of the PBC spectrum. In acoustic design, the imperfect coupling can be achieved by changing the number of tubes, as mentioned in Note 3.

Note 5. The experiment setup.

The flowchart of the experiment is shown in Fig. S7a. The signal generator downloads the excitation signal from computer, and then sends it to loudspeaker continuously. During the wave-matter interaction in the sample, the acoustic waves are measured by the microphone completely and sent to the oscilloscope after the pre-amplification. The excitation signal also works as a trigger to make the oscilloscope activate the function of recording. After averaging, the recording results will be sent to computer for post-process. The absorptive materials are glued to the inner wall of the sample to induce the loss modulation, as shown in Fig. S7b. We change the length of the absorptive materials to control the amount of loss introduced.

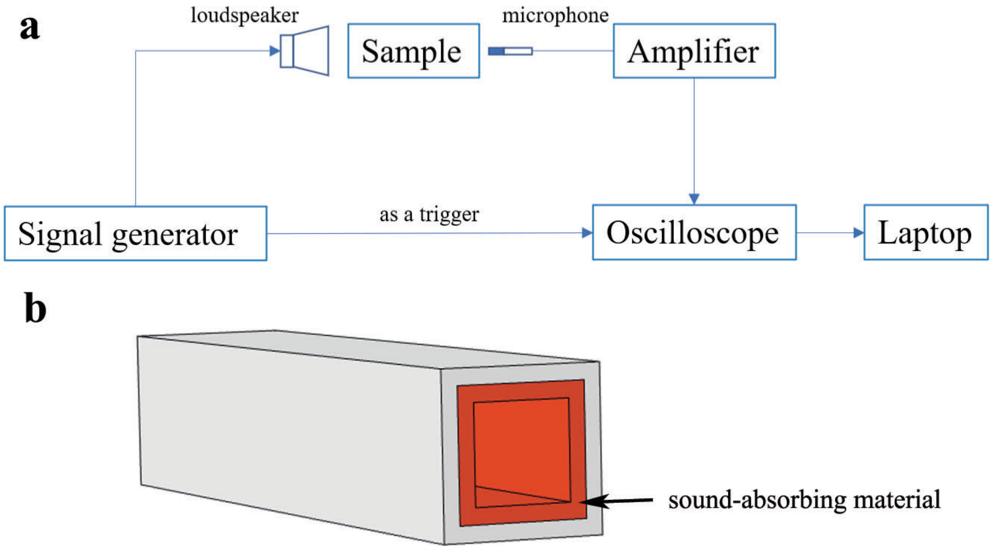

Fig. S7 Details of the experimental setup. a Illustration of the experimental setup. **b** Implementation of the sound-absorbing materials.

Note 6. List of supplementary videos.

Supplementary movie 1: Movie for acoustic field evolution of left-moving case.
Supplementary movie 2: Movie for acoustic field evolution of right-moving case.
