# Supplementary material for: Transient non-Hermitian skin effect
Source: Nat Commun. 2022 Dec 12;13:7668. doi: 10.1038/s41467-022-35448-2 (PMC9744917; doi:10.1038/s41467-022-35448-2)
Supplement: Supplementary file 3 — Description to Additional Supplementary Information [file 41467_2022_35448_MOESM3_ESM.pdf]

Description of Additional Supplementary Files

Supplementary movie 1: Movie for acoustic field evolution of left-moving case.

Supplementary movie 2: Movie for acoustic field evolution of right-moving case.
